# The Defense of Theory

**Published:** 1897-02

**Authors:** J. S. Cassidy

**Affiliations:** Covington, Ky.


### The Defence of Theory.

BY J. S. CASSIDY, COVINGTON, KY.

Read before the Ohio State Dental Society, at Columbus, December 3rd, 1896.

The object of this paper is to enter a mild protest against the habit we have acquired, during the last few years, of condemning all ideas not proven as true by actual experiment, and not for the purpose of suggesting anything of either a scientific or practical nature.

To introduce such an unorthodox text to such a body as this is presuming a good deal ; no one can realize how much until he finds himself in this position, but when one is constantly surrounded by an inelastic atmosphere of science, and affected, as we all are, by the grinding monotony of daily practice, a departure from the usual routine may not be inexcusable.

How often, alas! we have heard and read the ungrateful expression, "Give us more of the useful, and less of these fine-spun theories." At the same time, he who occasionally ventures to disobey this injunction is probably far more practical in his daily life than are his critics, and of this fact he is usually proud ; indeed, it seems that we resent the imputation as though it were a stigma of being too visionary rather than too practical. To use the oft-repeated words of Mr. Venus, in "Our Mutual Friend": "She did not wish to so regard herself, nor yet to be so regarded." Any statement that you do not indorse falls to the ground as unworthy of respect if you only say, "It is a mere theory," but a good thought is never lost, for truth is eternal. Imagination is the offspring of intelligence; and imagination, guided by philosophy, develops thought, which controls the evolution of theories that in time result in practical application, and, on the other hand, explain the existence of well-known phenomena by processes of reasoning admittedly correct. A long time may elapse from the setting forth of an idea and the verification of its value.

Columbus evidently struggled with his so-called vagaries a long time before a few friends were found to believe in him, and able to furnish him with means to consummate his tremendous project of either finding a new route to the Indies or of introducing a new world to the old, whereby we are granted the privilege of meeting pleasantly together on this occasion, in this beautiful city named in his honor.

At the time that Franklin dreamed of the lightning, and discovered its identity, circumstances were not such as to demand the uses to which it is put to-day. Perpetual motion, undoubtedly a fanciful figment of unbalanced minds, is, nevertheless, practically at work by the overflowing waters of Niagara, and who, indeed, can laugh at any one who says the future will witness the winds and waters of the earth judiciously harnessed, thus supplying heat and light and power illimitable.

Again, there may be a persistent demand for something needed as with anæsthesia, which, from time immemorial was hoped for, and although the means for which were pointed by out Davy in his original description of $N_2$. O., the idea remained in abeyance for nearly forty years, until Wells realised and put in practice the blessed inspiration. The atomic-theory of Dalton marked the birth of chemistry as a science, albeit the art had been practiced even from the time of Tubal Cain. This theory of Dalton has been essentially improved and accepted by all, and yet it is not proven as absolutely correct. It is a means to an end, correctly solving the problem of chemical affinities and the mathematical molecular construction of the most complex substances.

When Lavoisier put forth his theory of ordinary combustion and described the important part which he believed the newly-discovered element, oxygen, played in the field of natural phenomena, his views received little encouragement until Faraday proved, in detail, and beyond question, the nature of this wonderful reaction. If no other useful outcome could be traced to Lavoisier's theory than the oxy-hydrogen flame, such an example should be sufficient to render the modern scoffers of embryonic thought less skeptical in their opinions.

Was not the clear cut theory of Watt, which held that dental decay was caused by chemical agents produced only at the point of injury by a process now known as fermentation, a logical antecedent of Miller's experiments in that direction? Did not Miller theorize as to the genesis of this disease long before he produced, artificially, one of its varieties?

The imagination of Crookes, when he discovered radiant matter, the fourth state, about twenty years ago, conjured up the possibility of it being the borderland between material and spiritual entities—who can say that his was a spurious speculation in the light of what has already been accomplished by the Roentgen-ray?

We are a profession of neither realists nor idealists, but a happy combination of the two, and as such we are still, notwithstanding all our progress, unsatisfied with the materials we use for filling teeth. There is probably not one of us who has not devoted more or less serious thought to the coming ideal. Its coming, sometime, is surely not a "Will o' the Wisp" of the imagination; but, even if so, is it proper to discourage theorizing on such a subject?

Perhaps it is so, that life is too short and too full of pressing duties for the average dentist to devote much time to the consideration of questions belonging exclusively to pure science. Enough, possibly, for him to know is that certain causes will produce certain results; that, for instance, a certain base will neutralize a certain acid, resulting in the formation of a new body, which he knows in advance will possess certain properties; but why, really, it should be colorless, or blue or yellow, or hard or soft, are questions too trivial to engage his attention. Nevertheless, the minds of the best thinkers of to-day are tending to go beyond those evident causes and effects appreciable to our senses to the very depths of ultimate knowledge in the limitations of matter. This fact obtains more especially among those who know something of human physiology and pathology, whose life-work it is to combat disease. They assume that both metabolism and catabolism are processes of molecular, and, therefore, atomic interchange; and thus, also, with disease. The physical degeneration of a part, even though it be but temporary, whatever the cause, must proceed molecularly, and its restoration to health is governed by a similar process.

Where the part is permanently destroyed, as in dental caries, the lost portion must be restored by artificial means. Shall we consider a theory as mere "poppycock," which holds that absolutely-successful therapeutics in such cases will be the ideal filling, like in molecular motion with respect to heat like in color, like in hardness to the enamel itself, builded up molecule by molecule, through the influence of electrolysis or whatever the method may be named.

It is by the inherent properties of molecules only, in forming the mass, that we recognize matter in its various aspects; and if we are to keep in touch with the trend of therapeutics, and learn to know more of the nature of matter as we find it, we will be compelled to appeal more and more to our imagination in order to have a definite conception of molecules, at least, their individual construction by the chemical union of a given number and kind of atoms, and how these atoms, in homogeneous molecules, are similarly arranged.

We do not claim that theory is science, for it may be either true or false, and often is, unfortunately, when torn to nakedness, devoid of the elements of truth; but, nevertheless, each new discovery, marking the ascent of man to a higher civilization, was preceded by investigations governed by theories, and so it will be in the future. Theories, like the springs of running water which fructify the earth, should not be passed without good reason. They have not been, and will not be hereafter. To quote one of your profane and respected governors when referring to the proposed resumption of specie payment—"A d—d barren ideality."

